# Impact of habitual seaweed consumption on iodine nutrition and thyroid function: a non-randomized pre-post clinical study

**DOI:** 10.1007/s00394-025-03813-8

**Published:** 2026-01-16

**Authors:** Inger Aakre, Elinor Chelsom Vogt, Lene S Myrmel, Anne-Katrine Lundebye, Olivia Bysheim Helland, Sigrun Henjum, Lisbeth Dahl, Maria Wik Markhus, Synnøve Næss Sleire, Silje Jotun Løkken, Hanne Rosendahl-Riise

**Affiliations:** 1 Department of Seafood and Nutrition, Institute of Marine Research (IMR), 5817 Bergen, Norway; 2 Department of Medicine, Haukeland University Hospital, Bergen, Norway; 3 Department of Clinical Medicine, University of Bergen, Bergen, Norway; 4 Department of Nursing and Health Promotion, Oslo Metropolitan University, Oslo, Norway; 5 Center for Nutrition, Department of Clinical Medicine, University of Bergen, Bergen, Norway

**Keywords:** Iodine status, Thyroid function, TSH, Seaweed, Macroalgae

## Abstract

**Purpose:**

Macroalgae, also called seaweed, may serve as a dietary iodine source, although some species contain high iodine levels and may increase the risk of excessive intake. This non-randomized pre-post clinical trial aimed to assess iodine status, estimated iodine intake and thyroid function in habitual seaweed consumers (pre-intervention), and after a six-week cessation period of seaweed consumption (post-intervention).

**Methods:**

Habitual seaweed consumers (n = 49) were recruited by convenience sampling from the two largest cities in Norway. Urinary iodine concentration (UIC), urinary creatinine, iodine intake and thyroid function were assessed pre- and post-intervention. The thyroid function was assessed biochemically by measuring TSH, fT3, fT4 and TPO antibodies in serum. Seaweed intake was assessed using a seaweed specific food frequency questionnaire (FFQ).

**Results:**

The median (p25-p75) UIC and estimated iodine intake was 270 (185–970) µg/L and 658 (330–1516) µg/ day at pre-intervention and decreased substantially to 87 (52–138) µg/L and 189 (142–264) µg/ day post-intervention. Median estimated iodine intake pre-intervention exceeded the Tolerable Upper Intake Level (UL) for iodine, whereas post-intervention the estimated iodine intake was substantially decreased to well below the UL. The median serum TSH decreased significantly after cessation of seaweed consumption (from pre- to post-intervention). The largest decrease in TSH was seen in participants with the highest estimated iodine intakes pre-intervention.

**Conclusion:**

Ingestion of seaweed with high iodine content could potentially pose a risk of exceeding the tolerable upper intake level for iodine. Cessation of seaweed resulted in a significant decrease in TSH in this study.

**Supplementary Information:**

The online version contains supplementary material available at 10.1007/s00394-025-03813-8.

## Introduction

Macroalgae, also referred to as seaweed, have been historically consumed in many Asian countries. Seaweed as food has also entered the market in Europe and has become increasingly popular over the last decade [1, 2]. Sushi and seaweed salad are foods commonly known to most, however several different species are available for consumers including pure seaweed in flakes or shredded form, seaweed as ingredients in foods such as pasta, snacks and soups, or in dietary supplements [2, 3]. Many species of seaweed contain iodine and may therefore be a dietary source of this micronutrient [4, 5]. Globally, iodized salt is the most important iodine source, while other dietary sources of iodine primarily come from foods of animal origin [6]. Iodized salt is not mandatory in Norway, and the current level of iodine used in table salt is low (5 µg/g). Therefore, the most important iodine sources in the Norwegian diet are milk, dairy products and white marine fish [7]. Seaweed is therefore a potential iodine source, especially for individuals following plant-based diets.

Iodine is an essential micronutrient required to produce the thyroid hormones thyroxine (T4) and triiodothyronine (T3). Thyroid hormone receptors are found in cells throughout the body, and via gene transcription, thyroid hormones regulate a wide range of cellular and physiological functions essential for normal growth and development, neural differentiation, and metabolic regulation [8]. Iodine deficiency is one of the most common nutritional disorders worldwide. The most recent nationally representative dietary survey in Norway (“NORKOST 4”) showed that the iodine intake was adequate for men but below the recommended intake for women, when excluding supplements [9]. Furthermore, mild-to-moderate iodine deficiency has been reported in several population groups in Norway, including pregnant and lactating women [10, 11] as well as vegans and vegetarians [12, 13]. Iodine deficiency can cause inadequate thyroid hormone production resulting in increased pituitary secretion of thyroid stimulating hormone (TSH), causing thyroid gland hypertrophy and iodine deficiency goiter [14]. However, the relationship between iodine intake and thyroid disorders is u-shaped, and both iodine deficiency and excess may cause thyroid dysfunction [15]. Although iodine excess is generally well tolerated by most individuals, studies have shown that increased iodine intake over time, or abrupt changes in iodine intake can cause the thyroid gland to become overactive resulting in hyperthyroidism [16, 17]. Other studies, as summarized in a systematic review [18], have found that long term iodine excess is associated with subclinical hypothyroidism in different population groups, including adults, children and pregnant women. While the relationship between iodine excess and thyroid dysfunction is complex and not fully understood, some mechanisms have been proposed. Iodine-induced stimulation of the thyroid gland leading to hyperthyroidism is often referred to as the Jod-Basedow phenomenon and was described already in the 1800s [19]. Additionally, iodine-induced hypothyroidism can occur when a sudden increase in iodine intake inhibits thyroid hormone synthesis by downregulating iodine uptake and blocking the organification of iodide [20, 21]. This is referred to as the Wolff-Chaikoff effect and is often a transient state, where the hormone synthesis will be restored. However, in some cases, this may lead to an overproduction of thyroid hormones, resulting in hyperthyroidism. In other cases, the inhibitory effect may sustain, which may cause hypothyroidism and goiter [22, 23]. Evidence from epidemiological studies indicates that excessive iodine intake is more often related to hypothyroidism than hyperthyroidism [18]. The adequate intake of iodine established by the World Health organization as well as in the Nordic countries is 150 µg/day for adults [6, 24], and the tolerable upper intake level (UL) endorsed by the European Food Safety Authority (EFSA) is 600 µg/day for adults [25]. Thus, the optimal window of iodine intake is quite narrow.

In a recent exposure assessment by the EFSA, seaweed intake was found to pose a risk of excessive iodine intake among consumers [26], since many species of seaweed have very high iodine contents [27]. However, there is limited knowledge regarding the potential risks and benefits to human health from consuming seaweed in Europe. Studies from Japanese populations show that seaweed consumption can alter thyroid function [28, 29], though studies on the effect of excessive iodine intakes are limited and the evidence is still inconclusive [30]. A challenge in this research area is that randomized controlled trials (RCTs) providing seaweed species with high iodine content to participants may not be ethically appropriate due to the potential risks related to excessive iodine exposure.

In this study, we recruited participants that already habitually consumed seaweed to explore potential effects on iodine nutrition and thyroid function. The aim of this study was to assess iodine nutrition and thyroid function in habitual seaweed consumers, both before and after a six-week cessation period of seaweed consumption. Furthermore, we aimed to explore potential associations between iodine nutrition and thyroid function within this group.

## Methods

### Study design and recruitment

A non-randomized pre-post clinical trial was conducted from March 2022 until January 2023 in Norway. The purpose was to recruit habitual seaweed consumers to attend two study visits: pre-intervention (after habitual seaweed consumption) and post intervention (after cessation of seaweed consumption). The cessation period of seaweed consumption was six weeks. This period was based on the time patients receiving thyroxine treatment are expected to achieve a new equilibrium in thyroid function [31].

Healthy adults were recruited from Bergen and Oslo, the two largest cities in Norway, through convenience sampling. Inclusion criteria were adults ≥ 18 years of age, weekly seaweed consumption, and living in or around Oslo or Bergen. Exclusion criteria were pregnancy or lactation and known kidney disease. Participants with known thyroid disorders were excluded or evaluated for inclusion by an endocrinologist, depending on the state and stability of the condition.

The outcomes, iodine status and thyroid function, have not been thoroughly investigated in this group before, and the extent of variation within and between the outcomes remains unclear among seaweed consumers. Given that iodine intake is a known predictor of thyroid function, it was selected as the basis for calculating the sample size. The sample size was calculated based on iodine intake from macroalgae in a previous study involving 44 macroalgae consumers where the median urinary iodine concentration (UIC) was 1200 µg/L following habitual intake of macroalgae [32]. Estimated median iodine intake, excluding macroalgae, was 260 µg/day in this group. By assuming that 90% of iodine from food is excreted in urine and a urine volume of 1.5 L, this corresponds to a UIC of (260*0.9/1.5) 156 µg/L. The difference in UIC after macroalgae consumption and estimated UIC was 1200–156 = 1044 µg/L. The standard deviation for the difference was 2767 µg/L, which gives an effect size of 0.38 with the following calculation: Effect size = difference/SD of the difference. With a power of 80% this would require a sample size of 45 participants.

The study was advertised on social media, in a local newspaper, in various shops such as grocery and health food stores, and on websites selling seaweed products. Participants were also encouraged to invite others who were eligible for the study. Interested participants could sign up by e-mail, phone or through an online form accessed by a QR code. After participants showed interest, the first contact was made via email or phone, by a project worker who screened potential participants for eligibility. After inclusion, participants in the Bergen area received a consent form, a questionnaire, and equipment for urine sampling at home, prior to the first study visit. For participants living in and around Oslo, consent forms, questionnaires, and equipment for urine samples were sent by mail. The recruitment ended in January 2023, and the final sample consisted of 49 participants.

### Study procedures

Participants attended two study visits: pre-intervention and post-intervention (after the six-week cessation period). The participants were asked to refrain from seaweed both as food and as supplements during the six-week cessation period. Otherwise, there were no dietary restrictions. On the first study visit (pre-intervention), the participants were screened for their intake of iodine from foods other than macroalgae through a digital iodine-specific dietary screener [33] by a clinical dietitian. If participants were in danger of consuming inadequate amounts of iodine, due to exclusion of macroalgae, they were recommended to follow the advice from the Norwegian Directorate of Health, which is to eat white marine fish and drink milk to secure a sufficient intake of iodine, or to take a multimineral supplement [34]. In total, six participants reported at the second study visit that they had been taking an iodine containing supplement. The iodine doses from the supplements taken ranged from 75 to 500 µg/day. The study was administered by the Research Unit for Health Surveys (RUHS), a core facility at the University of Bergen and Haukeland University Hospital. The study visits in Bergen took place at RUHS, while the study visits in Oslo took place at Oslo Metropolitan University. Background information was collected pre-intervention, while dietary assessment, UIC and thyroid function were assessed at both pre- and post-intervention.

### Questionnaires

Demographic variables, such as age, height, body weight, education, and smoking habits, were reported by the participants through a questionnaire which also contained specific questions regarding the use of supplements and dietary patterns. A seaweed-specific non-quantitative Food Frequency Questionnaire (FFQ) was developed for this project, with the purpose of identifying seaweed species and food products with seaweed and at which frequency these were consumed. The FFQ included questions regarding specific macroalgae products such as fresh macroalgae, dried macroalgae, food products containing macroalgae as an ingredient and supplements with macroalgae. Since limited information exists about which seaweed species and products that are consumed, the FFQ included options for the participants to specify the species consumed, types of seaweed-containing foods and supplements. Furthermore, the FFQ included three open fields where participants could report products or foods not included in the questionnaire, if relevant. Participants could choose from the frequencies ‘rarely/never’, ‘less than once a week’, ‘1–3 times a week’, ‘4–6 times a week’, and ‘1–2 times a day’, and the recall period was six weeks. At the post-intervention visit, participants answered a questionnaire concerning compliance with the cessation of seaweed consumption, as well as questions regarding the duration of seaweed cessation and consumption of iodine-containing supplements during the study period.

Consumption of several different species was reported in the FFQ by participants, and the common names of these seaweeds will be used throughout the paper. An overview of the seaweed species common names, scientific names and group affiliation can be found in Table 1.

**Table 1:** Common name, scientific name, and group affiliation of the identified seaweed species from the FFQ in the study of Norwegian seaweed consumers (n = 49)

Common name	Scientific name	Group affiliation
Sugar kelp	*Saccarina latissima*	Brown
Bladder wrack	*Fucus vesiculosus*	
Oarweed	*Laminaria digitata*	
Winged kelp	*Alaria esculenta*	
Rock weed	*Ascophyllum nodosum*	
Kombu	*Saccharina spp*	
Sea spaghetti/ Thongweed	*Himanthalia elongata*	
Wakame	*Undaria pinnatifida*	
Hijiki	*Sargassum fusiforme*	
Sea lettuce	*Ulva lactuca*	Green
Gut weed	*Ulva intestinalis*	
Wrack siphon weed	*Vertebrata lanosa*	Red
Dulse	*Palmaria palmata*	
Nori	*Porphyra spp*	
Irish moss	*Chondrus crispus*	
Pink laver	*Porphyra umbilicalis*	
Clawed Fork Weed	*Furcellaria lumbricalis*	

### 24-h dietary recall

Two 24-h dietary recalls (24-HR) were conducted by a dietitian at both pre- and post-intervention to estimate the participants’ iodine intake from the diet. In total, four 24-HR were assessed for each participant, two at each time point, and an average of the two 24-HR was used for each time point to limit the within-person errors. One 24-HR at each time point was performed by telephone interview one to three days before the study visits, and the second 24-HR was performed in person at the study visits. We aimed to cover both one day of the weekend and one weekday. A picture booklet produced by the Norwegian Directorate of Health in the ‘Norkost 3’ survey was used to estimate portion size and amounts of foods consumed [35]. To estimate the daily iodine intake, the foods reported in the 24-HR were entered into an online dietary tool called “Kostholdsplanleggeren” (Diet planner), developed by the Norwegian Directorate of Health and the Norwegian Food Safety Authority [36], which uses food composition data from the Norwegian Food Composition Table. For foods missing in the dietary tool, efforts were made to find suitable replacements. Macroalgae and macroalgae products were not present in the dietary tool and were therefore not included in the calculations of estimated intakes. Consequently, the estimated iodine intake from the 24-HRs were used to estimate iodine intake from other dietary iodine sources than macroalgae.

### Urinary iodine concentration

Participants were asked to deliver six spot urine samples from six consecutive days preceding the pre- and post-intervention visits. Participants were provided with equipment (Vacuette urine system, Krensmünster, Austria) for collecting spot urine samples at home, which were then delivered at the study visits. A minimum of 15 mL urine sample was needed for analyses. Urine could be collected at any time during the day, except from the first morning void. Pre-analytical work was performed by transferring a urine aliquot (2 mL) from each spot sample into one pooled sample. The pooled urine samples were stored at -80ºC pending analysis. Urine samples were analyzed at the Institute of Marine Research (IMR) (accredited laboratory according to the ISO/IEC 17025 standard) by approved laboratory technicians. Iodine was determined by inductively coupled plasma-mass-spectrometry (ICP-MS) with an iCap Q ICP-MS equipped with an autosampler. The limit of quantification (LOQ) for urinary iodine was 7.8 μg/L. One sample was re-analyzed due to an extremely high iodine concentration, and since a similar result was found at retesting, this data was included. The trueness of the method was evaluated by analysis of certified reference material in the same run as the samples. The measurement uncertainty of the method was 20%, based on internal reproducibility and analysis of standard reference material..

### Creatinine in urine

Creatinine concentration in the urine samples was measured spectrophotometrically using the clinical analysis instrument Pentra C400 (Horiba ABX SAS, Montpellier, France 2019) at the IMR. The reagent used for the measurement was ABX Pentra Enzymatic Creatinine CP reagent (Horiba). Prior to the analysis, the reagent was controlled and calibrated using the Yumizen C Urine Level 1 and Level 2 controls (Horiba) and the MultiCal calibrator (Horiba). The measurement uncertainty (CV) was 2.5–2.6% for concentrations between 8000–20000 µmol/L, and 4.8% for samples below 8000 µmol/L.

### Data management of urinary iodine concentration and creatinine

UIC was adjusted for hydration using the urinary creatinine concentration (UCC), Eq. 1. The UIC: UCC ratio was used to estimate the daily urinary iodine excretion (UIE) for each of the participants using Eq. 2. The creatinine reference values used were retrieved from the VERA study [37]. We used the body weight related references (mmol/ kg/ day) for men and women according to different age groups. For men the following values were applied: 0.206 (20–29 y); 0.208 (30–39 y); 0.185 (40–49 y); 0.188 (50–59 y); 0.181 (60–69 y); 0.176 (70–79 y). For women the following values were applied: 0.181 (20–29 y); 0.183 (30–39 y); 0.178 (40–49 y); 0.158 (50–59 y); 0.149 (60–69 y); 0.142 (70–79 y). Each subject’s individual body weight was multiplied with the body weight related reference. To further estimate the daily iodine intake, we assumed a 92% iodine excretion in the urine Eq. 3 [38]. Iodine intake from macroalgae could not be assessed from the seaweed-specific FFQ used in the present study since amounts consumed were not reported. Therefore, we used the mean estimated iodine intake (EII) from the 24-HR, which did not include macroalgae consumption, and subtracted it from the mean estimated iodine intake from the urine samples, Eq. 4.

1 $$\mathrm{UIC\!:\!UCC \ ratio} \left( {\mu \mathrm{g}/ \mathrm{mmol}} \right) = \frac{ \mathrm{UIC} (\mu \mathrm{g}/\mathrm{L}) }{{\mathrm{UCC} \left( {\mathrm{mmol}/\mathrm{L}} \right)}} $$

2 $$\begin{aligned} & \mathrm{Estimated\;UIE}\left({\mu \mathrm{g}/\mathrm{day}}\right)\\ &= \frac{{\mathrm{UIC}(\mu \mathrm{g}/\mathrm{L})}}{{\mathrm{UCC}\left({\mathrm{mmol}/\mathrm{L}} \right)}}\cdot \mathrm{UCC \, ref.values}\left({\frac{{\frac{{\mathrm{mmol}}}{{\mathrm{kg}}}}}{{\mathrm{day}}}}\right)\\ &\quad \cdot \mathrm{body\;weight}\left({\mathrm{kg}}\right)\\\end{aligned}$$

3 $$\begin{aligned}&\displaystyle \textrm{Estimated iodine intake} \left({\mu \mathrm{g}/ \mathrm{day}} \right) \\ &\quad= \mathrm{UIE} (\mu \mathrm{g}/ \mathrm{day})/0.92\end{aligned}$$

4 $$\begin{aligned} &\displaystyle \mathrm{EII\,seaweed}\left({\mu \mathrm{g}/\mathrm{day}} \right)\, \\ & \quad= \,\,\mathrm{EII\,urine}\left({\mu \mathrm{g}/\mathrm{day}} \right) \,\\ &\qquad -\, \mathrm{EII\,24H\,recall}\left({\mu \mathrm{g}/\mathrm{day}} \right)\\\end{aligned}$$

### Thyroid function

Non-fasting blood samples were drawn both pre- and post-intervention using sterile disposable BD Vacutainer, Eclipse Blood Collection Needles and collected in 5 ml Vaquette tubes with gel (Serum Sep clot Activator with gel 5 ml). Sample contents were mixed by turning the tubes at least eight times and then left to coagulate for 30 to 120 min. Blood samples were subsequently centrifuged at 2000G, 20 °C, for 10 min and 1.5 ml of serum was transferred by pipette to a cryotube and stored at − 80 °C pending analysis. Serum was analyzed for TSH, free T3 (fT3), free T4 (fT4), thyroglobulin (Tg), thyroglobulin antibodies (TgAb), and thyroid peroxidase antibodies (TPOAb) at Haukeland University Hospital (certified laboratory NSEN-ISO 15189), by Electrochemiluminescence Immunoassay (ECLIA). For TSH, fT3, fT4, Tg, TgAb, and TPOAb the analytical variation/ and intraindividual biological variation was 5%/19%, 7%/7.9%, 5%/5.7%, 6.5%/13%, 6.4%/ 8.5%, and 8%/11.3%, respectively. An overview of the laboratory´s reference ranges is given in Table 2. We assessed thyroid function biochemically, in cooperation with an endocrinologist.

**Table 2:** Reference ranges used for thyroid function tests [56]

Blood constituents	Material	Method of analysis	Reference ranges
TSH (mIU/L)	Serum	ECLIA	0.4–4.5
Free T4 (pmol/L)	Serum	ECLIA	9.5–22.0
Free T3 (pmol/L)	Serum	ECLIA	3.1–6.8
Tg (µg /L)	Serum	ECLIA	NA ^a^
TgAb (kIU/L)	Serum	ECLIA	< 115 ^b^
TPOAb (kIU/L)	Serum	ECLIA	< 34

ECLIA, Electrochemiluminescence immunoassay

^a^ No reference values for Tg. ^b^TgAb values > 20 kIU/L may affect Tg concentrations

### Ethics

This study was conducted according to the guidelines laid down in the Declaration of Helsinki and all procedures involving human subjects were approved by the Regional Committee for Medical and Health Research Ethics in Norway (332,856) and registered at Sikt (Norwegian Agency for Shared Services in Education and Research; reference number 805910). Written informed consent was obtained from all subjects.

Participants were provided with information about the study in writing before participating and signed an informed consent form in order to be enrolled in the study. Enrolled participants could withdraw at any time without giving a reason. All project workers have completed Good Clinical Practice training. The study is registered at ClinicalTrial.org (NCT05940727).

### Statistics

SPSS version: 29.0.0.0 (IBM, Armonk, NY, USA) was used for data management and analysis. The primary outcome in this study was iodine nutrition and the secondary outcome was thyroid function. Descriptive analyses were done for all main variables (descriptives, iodine nutrition and thyroid function). Normality of the variables was assessed by visual inspection of histograms and QQ-plots. Due to skewed distributions of most variables and a small sample size, non-parametric measures were used to describe iodine nutrition and thyroid function, using median and p25-p75. For descriptive purposes, mean and standard deviations were also given. Differences in continuous variables between two categories were tested using the Mann Whitney U test.

Linear regression was used to explore determinants for TSH. Due to skewed data, TSH was log(10) transformed for the analysis. We assessed age, gender and BMI for associations with TSH log(10) pre-intervention using simple models. Furthermore, we explored whether TSH log(10) at pre-intervention was associated with Tg, UIC, and estimated iodine intake pre-intervention. Similarly, we explored whether TSH log(10) post-intervention was associated with Tg, UIC, and estimated iodine intake (post-intervention). For the post intervention data we additionally examined the association between TSH log(10) and number of days without seaweed consumption, as well as compliance with the study design. Compliance was assessed using a dichotomous variable indicating whether participants reported consuming macroalgae during the cessation period (yes/ no). Difference in TSH with duration of habitual seaweed intake ≤ one year and > one year, as well as 1–3 months and > three months were assessed using Mann Whitney U test.

## Results

### Participation flow and characteristics

**Fig. 1:**
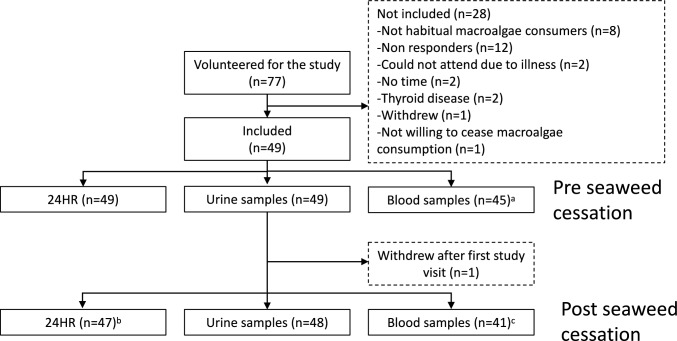
Participation flow and overview of the study sample and number of participants that delivered 24-h dietary recalls (24-HR), urine samples and blood samples. ^a^ Four missing due to practical reasons or illness. ^b^ One missing due to illness. ^c^ Seven missing due to practical reasons or illness

A total of 49 adult men and women were included in the study Fig. 1. The participants did not report any negative health effects during the study period. Adverse events related to thyroid function pre- or post-intervention, defined as deviations from laboratory references ranges were evaluated by an endocrinologist and communicated to the participants after their last study visit. Regarding compliance with the study design, 11 participants did not fully comply with the protocol by failing to exclude seaweed from their diet for the entire six-week period. They contacted the study personnel and were consequently scheduled for their second visit earlier than originally planned, because of non-compliance with the protocol. For these 11 participants, the second study visit was conducted after: 15 days (n = 1); 23 days (n = 1); 31 days (n = 1); 34 days (n = 1); 35 days (n = 4), 37 days (n = 1) and 38 days (n = 2). Furthermore, nine participants reported that they had consumed some seaweed or seaweed containing food during the cessation period. The amounts reported were occasional consumption and in small amounts, except from one participant who had consumed sushi consisting of maki rolls with nori 10 times. All participants were included in the analysis.

Pre-intervention characteristics of the participants are described in Table 3 and consisted of 73% women and 27% men. The mean ± SD age among the participants was 47 ± 14 years, and the minimum and maximum ages were 22 and 72 years. Forty-five percent reported following a specific diet or dietary pattern, and most of the participants (59%) had consumed macroalgae regularly for more than one year.

**Table 3:** Sample characteristics of Norwegian habitual seaweed consumers (n = 49)

Characteristics	
Gender n (%)	
Men	13 (27)
Women	36 (73)
Age, years	47 ± 14
BMI, kg/m^*2*^	24 ± 3.8
Normal weight (18.5–24.9 kg/m^2^), n (%)	32 (65)
Overweight (25.0–29.9 kg/m^2^), n (%)	14 (29)
Obese (≥ 30.0 kg/m^2^), n (%)	3 (6)
Birth country n (%)	
Norway	35 (71)
Other	14 (29)
Education n (%)	
High-school	13 (27)
1–4 years of higher education	13 (27)
> 4 years of higher education	23 (47)
Dietary patterns n (%)	
Pescatarian	3 (6)
Lacto-ovo vegetarian	2 (4)
Vegan	3 (6)
Other diet^b^	13 (27)
No specific	28 (57)
Duration of seaweed consumption^c^ n (%)	
1–3 months	12 (25)
3–6 months	5 (10)
6–12 months	3 (6)
> 1 year	29 (59)
Self-reported thyroid disease	
Hypothyroidism	3 (6)

Values are given as n (%) and mean ± SD

^a^ Other countries include: Japan, Slovakia, Malaysia, Thailand, South-Korea, Australia, Latvia, Denmark, Bosnia Hercegovina, Russia, Iceland. ^b^ Other diet: different dietary patterns mentioned, such as low carb diet, intermittent fasting, plant-based but including some animal food products, variations of a vegan diet (including honey, “wild” meat) and excluding gluten and dairy. ^c^ Duration of seaweed intake pre-study inclusion

### Seaweed intake

The total number of participants that reported intake of one or several species of pure fresh seaweed during the last six weeks from the seaweed specific FFQ was 25 (51%), and 29 (59%) reported intake of one or several species of pure dried seaweed Table 4. Data from the FFQ showed that in the fresh seaweed category, rockweed, wakame and sugar kelp were the most popular species, consumed by 22%, 12% and 5%, respectively. For the dried seaweed, sugar kelp (16%) and nori (12%) were most frequently consumed. Additionally, participants reported consuming foods which contained seaweed as an ingredient. The most consumed foods with seaweed were sushi, soups, and snacks. Spice mixes and smoothies containing seaweed were also quite commonly consumed among the participants. The spices included in the foods were recorded, however not presented in the table. The most common species in the food items containing seaweed as an ingredient, were nori and wakame. However, other species were reported: dulse, kombu, thongweed, Irish moss, winged kelp, bladder wrack, rockweed, pink laver, wrack siphon weed, tangle and unspecified species such as “kelp” and “mix”. Dietary supplements containing seaweed were consumed by 14% (n = 7) of the participants and 4 participants reported daily consumption Table 5.

**Table 4:** Frequency of intake of fresh and dried seaweed products during the last six weeks pre-intervention by study participants (n = 49)

**Pure seaweed**	Rarely ^d^		Less than once a week		1–3 times a week		4–6 times/ week		1–2 times/ day		Total among consumers	
**Fresh seaweed**	n	%	n	%	n	%	n	%	n	%	n	%
**Total**	**2**^**b**^	**4**	**12**^**b**^	**25**	**3**^**b**^	**6**	**11**^**b**^	**22**	**0**^**b**^	**0**	**25**^**c**^	**51**
Rock weed	0	–	0		0	–	11	22	0	–	11^a^	22
Wakame	1	2	5	10	0	–	0	–	0	–	6^a^	12
Sugar kelp	0	–	2	4	1	2	0	–	0	–	3^a^	6
Sea lettuce	0	–	1	2	1	2	0	–	0	–	2^a^	4
Winged kelp	0	–	2	4	0	-	0	–	0	–	2^a^	4
Dulce	0	–	1	2	1	2	0	–	0	–	2^a^	4
Unspecified	1	2	1	2	0	–	0	–	0	–	2^a^	4
Wrack siphon weed	0	–	2	4	0	–	0	–	0	–	2^a^	4
Bladder wrack	0	–	1	2	0	–	0	–	0	–	1^a^	2
Clawed Fork Weed	0	–	1	2	0	–	0	–	0	–	1^a^	2
Gut weed	0	–	1	2	0	–	0	–	0	–	1^a^	2
Thong weed	0	–			1	2	0	–	0	–	1^a^	2

**Dried seaweed**	n	%	n	%	n	%	n	%	n	%	n	%
**Total**	**1**^**b**^	**2**	**11**^**b**^	**22**	**19**^**b**^	**39**	**9**^**b**^	**18**	**4**^**b**^	**8**	**29 **^**c**^	**59**
Sugar kelp	0	–	2	4	4	8	2	4	0	–	8^a^	16
Nori	0	–	2	4	4	8	0	–	0	–	6^a^	12
Wrack siphon weed	1	2	4	8	1	2	0	–	0	–	6^a^	12
Dulce	0	–	3	6	2	4	1	2	0	-	6^a^	12
Pink laver	0	–	1	2	2	4	1	2	2	4	6^a^	12
Wakame	0	–	0	–	4	8	1	2	1	2	6^a^	12
Winged kelp	0	–	1	2	3	6	1	2	0	–	5^a^	10
Bladderwrack	0	–	0	–	2	4	2	4	0	–	4^a^	8
Sea lettuce	0	–	2	1	1	2	0	–	0	–	3^a^	6
Unspecified	0	–	0	–	1	2	1	2	1	2	3^a^	6
Rock weed	0	–	0	–	0	–	2	4	0	–	2^a^	4
Oar weed	0	–	0	–	0	–	2	4	0	–	2^a^	4
Kombu	0	–	1	2	0	–	1	2	0	–	2^a^	4
Hijiki	0	–	1	2	1	2	0	–	0	–	2^a^	4
Irish moss	0	–	0	–	1	2	0	–	0	–	1^a^	2
Thong weed	0	–	0	–	1	2	0	–	0	–	1^a^	2
Gutweed	0	–	1	2	0	–	0	–	0	–	1^a^	2

^a^ Total number of habitual consumers within each species. ^b^ Total number of participants that reported consumption of one or *several* species of fresh or dried seaweed within each frequency category the last 6 weeks. Different species could be reported by one participant; thus, the total number does not reflect the summarized numbers in each category of intake frequency. ^c^ Total number of consumers of either fresh or dry macroalgae, independent of frequency of intake. ^d^ Non consumers are not given

**Table 5:** Frequency of intake of food items with seaweed and seaweed containing dietary supplements by study participants during the last six weeks pre-intervention (n = 49)

Food with seaweed	Never/ rarely		Less than once a week		1–3 times a week		4–6 times/ week		1–2 times/ day		Total^a^	
	n	%	n	%	n	%	n	%	n	%	n	%
**Total**	**49**^**b**^	**100**	**36**^**b**^	**74**	**21**^**b**^	**43**	**12**^**b**^	**25**	**4**^**b**^	**8**	**43**^**c**^	**88**
Sushi	20	41	23	47	6	12	0	–	0	–	29^a^	59
Soup	26	53	13	27	5	10	5	10	0	–	23^a^	47
Snacks	35	71	10	20	3	6	1	2	0	–	14^a^	29
Spice mix	37	76	4	8	6	12	2	4	0	–	12^a^	25
Seaweed salt	38	78	5	10	1	2	3	6	2	4	11^a^	23
Smoothie	39	80	1	2	3	6	6	12	0	–	10^a^	20
Noodles	41	84	7	14	1	2	0	–	0	–	8^a^	16
Bakery products	43	88	5	10	0	–	0	–	1	2	6^a^	12
Stew	44	90	1	2	3	6	1	2	0	–	5^a^	10
Sweets	44	90	5	10	0	–	0	–	0	–	5^a^	10
Pickled/ fermented seaweed	45	92	3	6	1	2	0	–	1	2	4^a^	8
Pesto	47	96	0	–	0	–	2	4	0	–	2^a^	4
Sauce	47	96	0	–	2	4	0	–	0	–	2^a^	4
Caviar	48	98	1	2	0	–	0	–	0	–	1^a^	2
Meat replacement	47	98	2	4	0	–	0	–	0	–	2^a^	4
Jam	48	98	0	–	0	–	1	2	0	–	1^a^	2

Dietary supplements	n	%	n	%	n	%	n	%	n	%	n	%
**Total**	**1**	**2**	**1**	**2**	**1**	**2**	**0**	–	**4**	**8**	**7**^**c**^	**14**
Kelp supplements	0	–	1	2	0	–	0	–	1	2	2^a^	4
Oil from seaweed	0	-	0	–	0	–	0	–	2	4	2^a^	4
Channeled wrack supplements	1	2	0	–	0	–	0	–	0	–	1^a^	2
Mixed species supplements	0	–	0	–	1	2	0	–	0	–	1^a^	2
Bladderwrack supplements	0	–	0	–	0	–	0	–	1	2	1^a^	2

^a^ Total number of habitual (excluding rarely/ never) consumers within each food product. ^b^ Total number of participants that reported consumption of one or *several* foods with seaweed within each frequency category the last 6 weeks. Different foods could be reported by one participant; thus, the total number does not reflect the summarized numbers in each category of intake frequency. ^c^ Total number of consumers of foods or supplements with macroalgae, independent of frequency of intake

### Effect of intervention on iodine nutrition

**Fig. 2:**
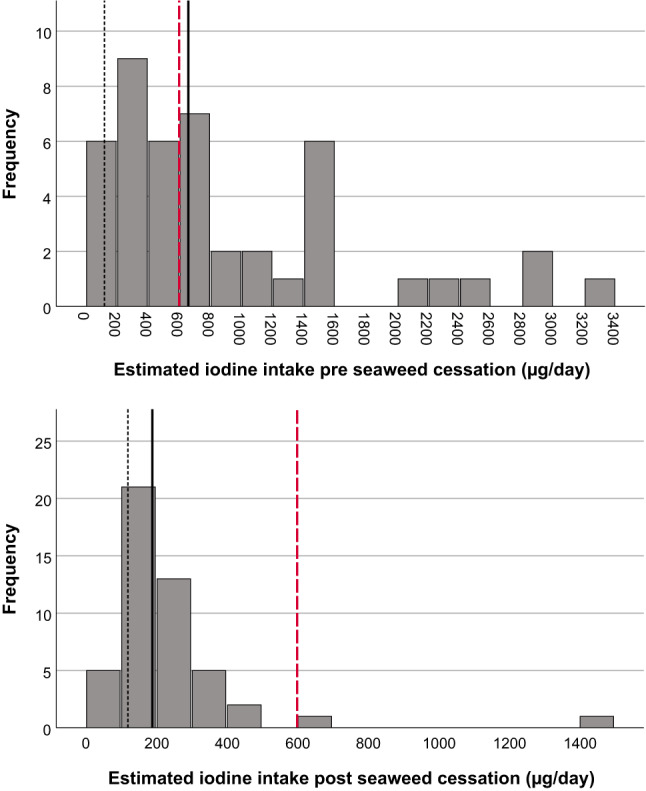
Estimated iodine intake of participants in the study based on urinary iodine concentration (Eq. 3) pre seaweed cessation (pre-intervention) (n = 45*) and post seaweed cessation (post-intervention) (n = 48). The red dotted line indicates the tolerable upper intake level set by EFSA [25]. The black dotted line indicates the provisional average requirement from the NNR 2023 [24]. The black solid line indicates the median estimated iodine intake. *Three participants with estimated iodine intake of above 5000 µg/day were excluded from the figure for visual reasons

The iodine nutrition of the participants at pre- and post-intervention is presented in Table 6. The UIC, UIC:UCC ratio, estimated UIE and estimated iodine intake from the UIC were substantially different between the two time points, and all the measures decreased significantly from pre- to post-intervention. As seen from the means and SDs, the data were highly skewed pre seaweed cessation. This is furthermore illustrated in Fig. 2. The estimated iodine intake from the 24HRs, which did not include seaweed intake, was similar at the two time points. The median (p25-p75) estimated iodine intake from seaweed alone was 381 (140–1381) µg/day pre seaweed cessation.

**Table 6:** Iodine nutrition in seaweed consumers pre-intervention (n = 49) and post-intervention (n = 48)

	Pre-intervention (n = 49)	Post-intervention (n = 48)	*P* difference ^e^
UIC *µg/L*			
Median (p25-p75)	270 (185–970)	87 (52–138)	< 0.001
Mean ± SD	1155 ± 3792	114 ± 133	
UCC *mmol/L*			
Median (p25-p75)	7.1 (4.7–10.6)	5.6 (3.4–10.5)	0.206
Mean ± SD	7.7 ± 3.9	6.7 ± 4.0	
UIC:UCC ratio^a^ *µg/mmol*			
Median (p25-p75)	51.5 (22.2–119)	14.4 (10.2–21.3)	< 0.001
Mean ± SD	156 ± 397	17.9 ± 12.7	
Estimated UIE^b^* µg/day*			
Median (p25-p75)	605 (303–1394)	174 (130–243)	< 0.001
Mean ± SD	1799 ± 3887	217 ± 192	
Estimated iodine intake^c^* µg/day*			
Median (p25-p75)	658 (330–1516)	189 (142–264)	< 0.001
Mean ± SD	1837 ± 4425	236 ± 209	
Estimated iodine intake from 24-HR dietary recall^f^ *µg/day*			
Median (p25-p75)	127 (71–321)	138 (61–719)	0.994
Mean ± SD	247 ± 282	224 ± 248	
Estimated iodine intake from seaweed^d^ *µg/day*			
Median (p25-p75)	381 (140–1318)	NA	
Mean ± SD	1597 ± 4236	NA	

^a^Eq. 1. ^b^Eq. 2.^c^Eq. 3. ^d^Eq. 4. ^e^ Mann Whitney U test for difference between pre- and post-intervention. ^f^ Mean of two 24-h dietary recalls at each time point, seaweed was not included. NA: Not available. UIC: Urinary Iodine Concentration. UCC: Urinary Creatinine Concentration. UIE: Urinary Iodine Excretion

**Fig. 3:**
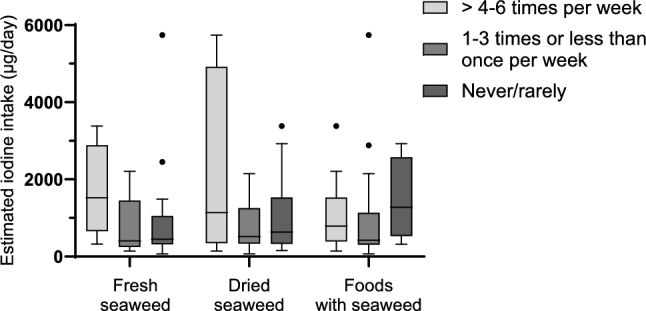
Estimated iodine intake pre seaweed cessation (pre-intervention) in different groups of seaweed intake frequencies. The boxes indicate the 25th and 75th percentiles, the horizontal line within the box is the median and whiskers were calculated using the Tukey method. The dots indicate outliers. Differences were tested between the two categories with lowest and highest frequencies by Mann Whitney U test, showing p values of 0.054 for fresh seaweed; 0.255 for dried seaweed; 0.541 for foods with seaweed. Six outliers were excluded from the plot for visual reasons

Stratified analysis categorized by seaweed intake frequency, revealed that participants that consumed fresh and dried pure seaweed more frequently had higher estimated iodine intake than those that consumed fresh and dried seaweed less often. Participants that reported consumption of fresh or dried seaweed > 4–6 times per week had a median (p25-p75) estimated iodine intake of 1524 (655–2882) µg/ day and 1139 (350–4919) µg/ day, respectively. Those that reported consumption of fresh or dried seaweed never or rarely had a median (p25-p75) estimated iodine intake of 447 (317–1045) µg/ day and 631 (329–1530) µg/ day. However, the differences were not statistically significant. For foods with seaweed as an ingredient and dietary supplements with seaweed, no such trend was seen (Fig. 3 and Supplementary Table 1).

We furthermore examined the total number of different species the participants had included in the diet. For fresh seaweed, three participants reported consuming three or more different species in the last six weeks, while for dried seaweed 11 participants reported consuming three or more different species (13 participants combined since one was in both categories). Among these 13 participants that had consumed three or more species of either fresh or dried seaweed the last 6 weeks, the median (p25-p75) estimated iodine intake was 910 (509–3973) µg/day, while for those that had consumed less than three different species, the estimated iodine intake was 447 (319–1470) µg/day. For the foods with seaweed as an ingredient, eight participants reported consumption of three or more foods with seaweed 1–3 times per week or more often. Among these, the median (p25-p75) estimated iodine intake was 1299 (686–2191) µg/day, while the participants that reported consumption of less than three different foods per week with seaweed in the last six weeks had an estimated iodine intake of 524 (318–1453) µg/day. Since the iodine content varies greatly among species of seaweed, consumers of brown seaweed species with known high iodine content [39] were selected and intake of these species were examined. Eleven participants reported eating these species, oar weed, sugar kelp and kombu, in variable frequencies. The median (p25-p75) estimated iodine intake among consumers of these brown seaweed species was 1490 (702–5740) µg/day, compared to 447 (317–1239) µg/day among consumers of all other species (data not shown).

### Effect of intervention on thyroid function

**Fig. 4:**
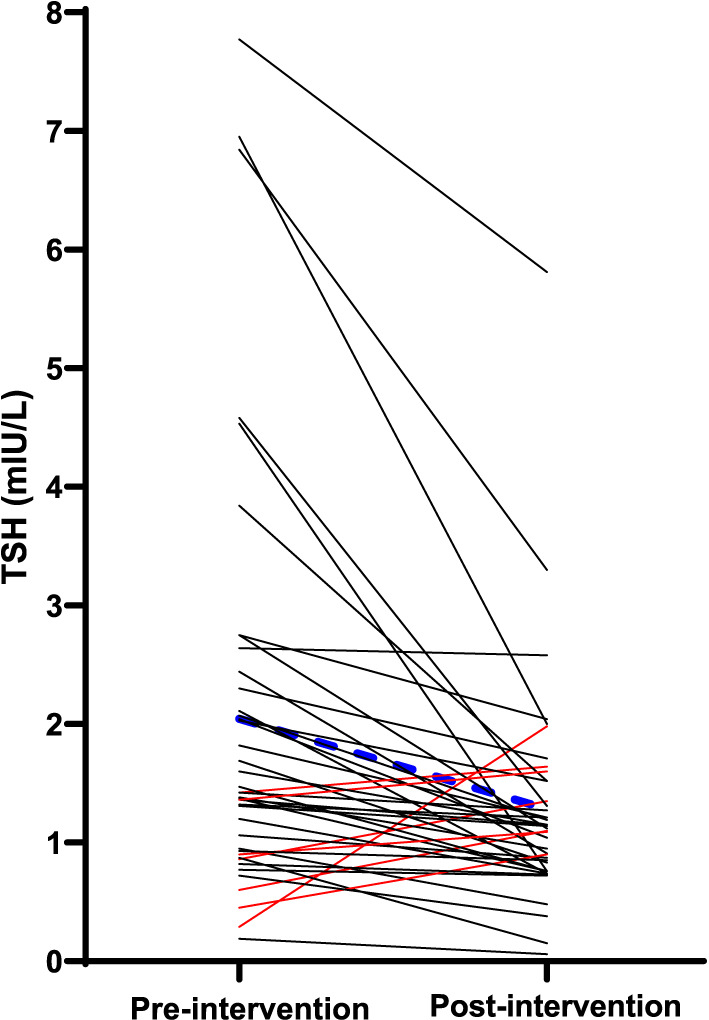
TSH (mIU/L) in participants after habitual seaweed consumption (pre-intervention) and after cessation of seaweed consumption (post-intervention). Seven individuals had an increase in TSH indicated by the red lines. 34 individuals had a decrease in TSH, indicated by the black lines. The thick blue dotted line indicates the mean

A decrease in TSH was seen from pre- to post-intervention among the participants. No substantial changes were observed in fT4 and fT3 levels between the two time points Table 7. Pre-intervention, five participants had elevated TSH (subclinical hypothyroidism) with one of them still showing elevated levels post-intervention. Two participants had low TSH at pre-intervention, and one of them remained low post-intervention. Among the participants with normal thyroid function pre-intervention, two showed elevated TSH post-intervention. All participants with elevated TSH had TSH < 10 mIU/L (Supplementary Table 2). Of the participants with blood samples at both time points (n = 41), 14% had an increase in TSH and 86% had a decrease from pre to post cessation of seaweed Fig. 4.

**Table 7:** Thyroid function tests among the participants after habitual seaweed consumption (pre intervention) and after cessation of seaweed intake (post intervention)

	Pre-intervention (n = 45)	Post-intervention (n = 41)	*P*^a^	Ref.^b^
TSH *mIU/L*				0.4–4.5
Median (p25-p75)	1.4 (0.9–2.5)	1.1 (0.8–2.0)	0.016	
Mean ± SD	2.1 ± 1.7	1.3 ± 1.0		
fT4 *pmol/L*				9.5–22.0
Median (p25-p75)	15.3 (13.7–16.4)	15.2 (14.1–16.0)	0.866	
Mean ± SD	15.0 ± 2.0	14.9 ± 1.8		
fT3 *pmol/L*				3.1–6.8
Median (p25-p75)	4.4 (3.9–4.9)	4.5 (4.1–4.8)	0.962	
Mean ± SD	4.5 ± 0.8	4.5 ± 0.6		
TPOAb *kIU/L*				< 34
Positive n (%)	4 (9.0)	2 (4.9)		
TgAb *kIU/L*				115
Positive n (%)	2 (4.9)	4 (9.8)		
Tg *µg/L*				
Median (p25-p75)	16.4 (11.2–31-0)	16.9 (10.2–27.5)	0.892	
Mean ± SD	24.4 ± 22.8	21.3 ± 16.9		

^a^ Tested with Mann Whitney U test. ^b^Laboratory reference ranges [55]

Different predictors for TSH log(10) pre-intervention were furthermore explored in linear regression models. Tg (p = 0.012), UIC (p = 0.021), and estimated iodine intake (p = 0.022) were all significantly associated with TSH log(10) in simple linear regression models. Neither gender (p = 0.365), age (p = 0.177), nor BMI (p = 0.480) were significantly associated with TSH log(10) pre-intervention. Post-intervention, no statistically significant associations were seen between TSH log(10) and number of days without seaweed (p = 0.610), compliance with study design (p = 0.640), Tg (p = 0.525), UIC (p = 0.788), or estimated iodine intake (p = 0.972).

### Iodine intake and thyroid function

Tertiles were used to group UIC, estimated iodine intake, and Tg, to explore the thyroid function markers TSH, fT4 and fT3 in the different groups of iodine nutrition. For TSH pre-intervention, there was a steady increase as the iodine intake increased. Furthermore, there was a small decrease in fT4 and fT3 with increasing iodine intake pre-intervention Table 8. The same association between iodine intake and TSH was not seen post-intervention (Supplementary Table 3). Neither did we see an association between iodine nutrition pre-intervention on thyroid function tests post-intervention (Supplementary Table 4).

**Table 8:** TSH, fT4 and fT3 in different groups of iodine status, estimated iodine intake and thyroglobulin levels pre-intervention

	TSH *mIU/L* (n = 45)	p ^a^	fT4 *pmol/L* (n = 45)	p ^a^	fT3 *pmol/L* (n = 45)	p ^a^	TPOAb positive (n = 45)
Iodine nutrition pre seaweed cessation							
UIC, *µg/L*							
< p33 (200 *µg/L)*	1.4 (0.8–1.8)	0.093	15.1 (13.5–16.4)	0.448	4.3 (3.9–4.9)	0.552	1 (2)
p33-p66 (200–490 *µg/L)*	1.3 (0.9–2.6)		15.6 (14.4–17.1)		4.6 (4.3–5.4)		2 (4)
> p66 (490* µg/L)*	1.8 (1.2–4.0)		14.0 (12.9–16.1)		4.1 (3.9–4.6)		1 (2)
Estimated iodine intake^c^ *µg/day*							
< p33 (407 *µg/day)*	0.9 (0.8–1.4)	< 0.001	15.3 (14.2–17.2)	0.217	4.6 (4.0–5.1)	0.158	0
p33-p66 (407–1155 *µg/day)*	1.4 (0.8–2.6)		15.3 (13.1–16.2)		4.5 (4.0–4.8)		2 (4)
> p66 (1155 *µg/day)*	2.4 (1.5–4.0)		14.2 (12.9–16.0)		4.1 (3.9–4.5)		2 (4)
Tg *µg/L*							
< p33 (12.7 *µg/L)*	1.4 (0.9–1.7)	0.067	15.3 (13.7–16.3)	0.935	4.4 (4.0–4.8)	0.233	0
p33-p66 (12.7–21.4 *µg/L)*	1.3 (0.7–2.8)		15.6 (13.1–17.4)		4.7 (4.1–5.1)		2 (4)
> p66 (21.4 *µg/L)*	2.0 (1.1–4.6)		14.8 (13.9–16.2)		4.1 (3.8–4.6)		2 (4)

Values are given as median (p25-p75) and n (%) of the total sample (n = 45).^a^ Differences were tested only between the lowest and highest tertiles (p33 and p66) with Mann Whitney U test. ^b^Eq. 2.^c^Eq. 3. < p33: tertile 1; p33-p66 = tertile 2; > p66 = tertile 3

**Fig. 5:**
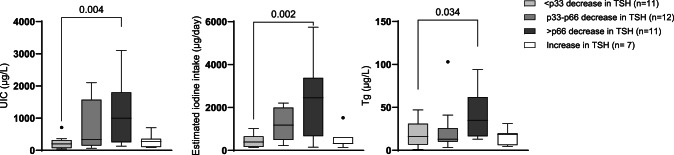
Iodine nutrition pre-intervention in participants with decrease in TSH (n = 34) from pre- to post-intervention divided into tertiles, and with an increase in TSH (n = 7) from pre- to post-intervention. The boxes indicate the 25th and 75th percentiles, the horizontal line within the box is the median and whiskers were calculated using the Tukey method. The dots indicate outliers. The different percentiles for decrease in TSH were: Tertile 1 (< p33): 0.4 mIU/L; tertile 2 (p33-p66): 0.4–0.9 mIU/L; tertile 3 (> p66): 0.9 mIU/L. Differences tested with Mann Whitney U test between the two categories Tertile 1 (< p33) and Tertile 3 (> p66). Two outliers were excluded from the plot for visual reasons

The mean difference in TSH from pre- to post-intervention in the participants with a decrease in TSH (n = 34) was 0.6 mIU/L. As seen from Fig. 5 and Supplementary Table 5, the participants with the largest decrease in TSH from pre- to post-intervention had higher UIC and estimated iodine intake pre-intervention. For the participants with the smallest decrease in TSH, the median UIC and estimated iodine intake were 200 µg/L and 395 µg/ day, respectively. While for those with the largest decrease in TSH, the median UIC and estimated iodine intake were 1000 µg/L and 2454 µg/ day, respectively. There was a substantial difference observed in Tg, and participants with the smallest decrease in TSH had lower Tg pre-intervention (16.2 µg/L), whereas those with the largest decrease in TSH had higher Tg (35.0 µg/L) pre-intervention. There were seven participants with an increase in TSH from pre- to post-intervention. The median UIC and estimated iodine intake were 86 µg/L and 322 µg/ day, respectively, for those with an increase in TSH which was substantially lower than for those with a decrease in TSH. We assessed whether there was a difference in TSH pre-intervention based on the duration of habitual seaweed consumption. There was no statistically significant difference in TSH between duration ≤ one year and > one year (p = 0.862) or with a duration of 1–3 months and > three months (p = 0.441).

## Discussion

In this non-randomized pre-post clinical trial of habitual seaweed consumers from Norway, the median estimated iodine intake pre-intervention exceeded the UL for iodine of 600 µg/ day. After the six-week intervention period with cessation of seaweed in the diet, the estimated iodine intake was substantially decreased to median (p25-p75) 189 (142–264) µg/ day, well below the UL for iodine. The median serum TSH decreased significantly from pre-intervention to post-intervention (after cessation of seaweed consumption). The participants with the largest decrease in TSH also had the highest estimated iodine intakes pre seaweed cessation. For fT3 and fT4 a slight and non-significant decrease was observed.

We found that the frequency of seaweed consumed, and the species included, varied widely among the participants. Seaweed has been regarded a niche food in many European countries, however recent studies from France, UK, Netherlands, Portugal, and Norway suggest that seaweed is becoming a more commercially available food [1–3, 40]. In this study, the large variety of species and food items consumed suggests that seaweed may be widespread beyond a niche food.

The median estimated iodine intake pre seaweed cessation (658 µg/day) exceeded the UL of 600 µg/day. The estimated iodine intake in this study was substantially higher than the findings of a recent nationally representative dietary survey in Norway, which reported a mean intake of 186 µg/ day for men and 163 µg/ day for women [9]. Given that we found consistent estimated iodine intake from 24-HRs, excluding seaweed, across both time points in this study, we suggest that the elevated iodine intake could be explained by the consumption of seaweed. Our suggestion is further supported by the significant decrease in UIC after cessation of seaweed consumption. Excessive iodine intake has previously been observed after seaweed consumption in Norway [32], Japan and Korea [41, 42]. Seaweed refers to a range of different species with different iodine content. Species with low iodine content, consumed in an adequate amount could serve as a dietary iodine source, which could be important for iodine deficient populations or for individuals on a vegan or vegetarian diet that lack dietary iodine sources. To achieve this, labeling the iodine content of commercially available seaweed products is required, to secure reliable and safe products for all consumer groups.

Iodine excess might lead to thyroid disturbances, though the evidence is not fully consistent. The baseline median TSH in this study was 1.4 mIU/L, which is very similar to what has been found in a healthy Norwegian female population, however the 75th percentile was higher in this study than in the reference population [43]. At group level, the participants in the present study had a decrease in TSH after cessation of seaweed consumption. Several observational studies have found associations with excessive iodine intake and elevated TSH concentrations [44–46], however evidence is conflicting, and others have not seen any effect of excessive long term iodine intake. In a large multicenter study from East Africa, 4636 participants from different life stage groups had UIC either above the WHO thresholds of adequate iodine nutrition, or above the threshold for excessive iodine nutrition. All population groups had elevated Tg concentrations, but no associations were seen for thyroid function [47]. Only a few RCTs have been conducted to assess possible effects of iodine excess on thyroid function. A Japanese double blind RCT (n = 104) investigated the effect of seaweed supplements containing 1.2 mg iodine given daily for eight weeks where the primary outcome was lipid metabolism. As a secondary outcome TSH was assessed, and a significant increase in TSH was seen for both men and women in the intervention group [48]. Similarly, seaweed supplements containing 475 µg were given daily to American postmenopausal women (n = 25) for seven weeks, and a slight but significant increase in TSH was observed after the intervention [49]. In a RCT from China with 256 healthy adults, participants were assigned to different iodine supplementation groups to explore the safe intake level of iodine, where the iodine doses ranged from 0 to 2000 µg/ day. The iodine intake was associated with TSH, and a larger increase in TSH was seen in the groups with the higher iodine supplementation [50]. Similar results were observed in the current study, as TSH levels were substantially and significantly higher in the group with the highest estimated iodine intake pre seaweed cessation (Table 8). No changes were seen in fT3 and fT4 from pre- to post intervention. A small, insignificant decrease was seen with higher iodine intakes when divided by different levels of iodine intake pre-intervention. Serum TSH levels are considerably more sensitive than direct thyroid hormone measurements for assessing the pituitary–thyroid axis, and small changes in free T4 can result in large changes in serum TSH levels [51].

Looking only at the participants with a decrease in TSH from pre- to post-intervention, we observed a clear association between estimated iodine intake and TSH, where the participants with the lowest decrease in TSH had a median (p25-p75) estimated iodine intake of 395 (163–655) µg/day, and the participants with the largest decrease in TSH from pre- to post-intervention had an initial median (p25-p75) estimated iodine intake of 2454 (667–3382) µg/day. The median (p25-p75) estimated iodine intake for the participants with an increase in TSH from pre- to post-intervention was 322 (317–623). These findings indicate that excessive iodine intakes are associated with higher TSH levels.

Even though the TSH levels decreased in most individuals after they stopped eating seaweed, the decrease was modest for most participants, and within the laboratory reference range. A literature review assessing the variation in TSH, found that variations even within reference ranges was associated with adverse health outcomes [52]. Higher TSH levels were associated with cardiovascular risk factors, such as blood pressure, blood lipid levels and mortality from coronary heart disease; metabolic parameters such as weight, BMI, and metabolic syndrome; and pregnancy outcomes such as spontaneous pregnancy loss. According to the authors, the evidence for all the mentioned outcomes was considered good, being derived from large cohort studies [52]. However, treatment of subclinical hypothyroidism is a debated topic, since long-term RCTs showing a positive effect of treatment are limited [53, 54]. In our study, a possible effect on TSH was seen, although it is not clear whether this is of any clinical relevance to this population. However, individuals with the above-mentioned comorbidities might be in a higher risk group than others. Furthermore, pregnant and lactating women, children [30], elderly and people with autoimmune thyroid disorders [55] are considered vulnerable to excessive iodine intakes and or changes in TSH. Therefore, inclusion of seaweed species with high iodine levels could pose a possible health risk for these population groups.

### Strengths and limitations

This is the first study to assess possible effects of cessation of habitual seaweed consumption on thyroid function. In this study, six repeated urine samples were collected at both time points (pre- and post-intervention) from each individual, which limits the random errors in UIC. We furthermore used weight and sex related creatinine references to adjust the UIC for hydration status. However, the estimation of UIE assumes similar urine volume in the spot samples and the reference values, which can be considered a weakness. Furthermore, when calculating the iodine intake from urine, we assumed a 92% excretion of iodine in urine, which is an estimate. The estimated iodine intake from seaweeds were calculated by subtracting the estimated iodine intake from the diet (excluding seaweed) by 24HRs from the estimated iodine intake calculated from UIC, which are two different methods, where the first is a dietary assessment method and the other serves as a biomarker. The estimated iodine intake from seaweed should therefore be considered a crude estimate. Bioavailability is often an issue when considering iodine uptake from seaweed [27], in the present study iodine nutrition was assessed using biomarkers thereby integrating bioavailability.

For various reasons not all the participants complied with the study design, which is a weakness in this study. However, we did not find any associations between the number of days without seaweed or compliance with seaweed cessation and TSH, which may indicate that the robustness of the observed association between TSH and iodine nutrition is strengthened. Finally, this study had limitations in the sample size and a heterogenous study population in terms of seaweed intake (species consumed and frequency), which may have increased the probability of type 2 errors. However, a decrease in TSH after cessation of seaweed consumption was still observed, as well as an association between TSH and increasing iodine intakes. This is the first study of its kind, and we believe these results need to be further investigated in larger and more robust studies, e.g. a multicenter RCT, recruiting diverse participants and with follow-up at several time-points, including a long-term follow-up to assess whether potential effects would last or diminish over time.

## Conclusion

Seaweed has become a commercially available food and is increasing in popularity. This is the first study to assess the effects of deprivation of seaweed in a group of habitual seaweed consumers. Iodine intake from seaweed was associated with changes in TSH levels in this study. We found that the estimated iodine intake was variable among the habitual seaweed consumers, but the median intake after habitual seaweed consumption was excessive. Seaweed refers to a range of different species, with different iodine content. In conclusion, consumption of seaweed with high iodine contents could pose a risk of consumers exceeding the Tolerable Upper Intake Level for iodine. Exclusion of seaweed from the diet decreased TSH slightly, but significantly in this group.

## Supplementary Information

Below is the link to the electronic supplementary material.

Supplementary file 1 (DOCX 32 KB).

## Data Availability

Aggregated and anonymized data are available from thecorresponding author upon reasonable request.
