# Supplementary material for: Impact of habitual seaweed consumption on iodine nutrition and thyroid function: a non-randomized pre-post clinical study
Source: Eur J Nutr. 2026 Jan 16;65(1):27. doi: 10.1007/s00394-025-03813-8 (PMC12811364; doi:10.1007/s00394-025-03813-8)
Supplement: Supplementary file 1 — (DOCX 32 KB). [file 394_2025_3813_MOESM1_ESM.docx]

**Supplementary tables**

**Impact of habitual seaweed consumption on iodine nutrition and thyroid function**

**- A non-randomized pre-post clinical study**

Inger Aakre ^1*^, Elinor Chelsom Vogt ^2^, Lene Secher Myrmel ^1^, Anne-Katrine Lundebye ^1^, Olivia Bysheim Helland ^3^, Sigrun Henjum ^4^, Lisbeth Dahl ^1^, Maria Wik Markhus ^1^, Synnøve Næss Sleire ^1^, Silje Jotun Løkken ^5^, Hanne Rosendahl-Riise ^5^

^1^ Department of Seafood and Nutrition, Institute of Marine Research, Bergen, Norway

^2^ Department of Medicine, Haukeland University Hospital, Bergen, Norway

^3^ Department of Clinical Medicine, University of Bergen, Bergen, Norway

^4^ Department of Nursing and Health Promotion, Oslo Metropolitan University, Oslo, Norway

^5^ Center for Nutrition, Department of Clinical Medicine, University of Bergen, Norway

***Corresponding author:** Inger Aakre, Institute of Marine Research (IMR), 5817 Bergen, Norway; e-mail: [inger.aakre@hi.no](mailto:inger.aakre@hi.no); Phone: +47 48132574

**Table S1** Iodine nutrition pre seaweed cessation in different groups of seaweed intake frequencies

| Intake frequencies^a^ | Estimated iodine intake^b^ *µg/day* | *P*^c^ |
| --- | --- | --- |
| Fresh seaweed |  |  |
| > 4-6 times per week (n=11) | 1524 (655-2882) | 0.054 |
| 1-3 times or less than once per week (n=12) | 404 (248-1453) |  |
| Never/rarely (n=25) | 447 (317-1045) |  |
| Dried seaweed |  |  |
| > 4-6 times per week (n=12) | 1139 (350-4919) | 0.255 |
| 1-3 times or less than once per week (n=16) | 516 (331-1252) |  |
| Never/rarely (n=20) | 631 (329-1530) |  |
| Foods with seaweed as an ingredient |  |  |
| > 4-6 times per week (n=16) | 789 (389-1530) | 0.541 |
| 1-3 times or less than once per week (n=26) | 421 (306-1134) |  |
| Never/rarely (n=6) | 1278 (531-2573) |  |
| Dietary supplements with seaweed |  |  |
| Yes (n=7) | 703 (361-1536) | 0.155 |
| No (n=41) | 444 (152-660) |  |

^a^ If a consumer had reported different intake frequencies for several species, the highest was selected. ^b^Eq.3. ^c^ Differences were tested between the two categories with lowest and highest frequencies by Mann Whitney U test, except from supplements where ‘yes’ and ‘no’ were the categories tested.

**Table S2 1** Development of biochemically assessed thyroid function among the participants after habitual seaweed consumption (pre-intervention) and after cessation of seaweed consumption (post-intervention).

| Pre seaweed cessation | *n* |  | Post seaweed cessation | *n* |
| --- | --- | --- | --- | --- |
| **Hypothyroidism** |  |  |  |  |
| Subclinical hypothyroidism ^a^ | *5* |  | Subclinical hypothyroidism ^b^ | *1* |
|  |  |  | Normal function | *4* |
| **Hyperthyroidism** |  |  |  |  |
| Subclinical hyperthyroidism ^c^ | *2* |  | Subclinical hypothyroidism | *1* |
|  |  |  | Normal function ^d^ | *1* |
| **Normal thyroid function** ^e^ | *38* |  | Subclinical hypothyroidism  *^f^* | *2* |

^a^ Of which one were TPOAb positive. ^b^ TPOAb positive.^c^ One TgAb pos. ^d^ TgAb pos. ^e^3 were TPOAb pos and 2 were TgAb pos.^f^ One were TgAb pos. The TSH values pre cessation for these two individuals were 0.72 and 0.87 mIU/L.

**Table S3** TSH, fT4 and fT3 post-intervention in different groups of iodine status and estimated iodine intake post-intervention.

|  | TSH post-intervention  *mIU/L* (n=41) | p ^a^ | fT4 post-intervention  *pmol/L* (n=41) | p ^a^ | fT3 post-intervention  *pmol/L* (n=41) | p ^a^ | TPOAb positive  Post-intervention (n=41) |
| --- | --- | --- | --- | --- | --- | --- | --- |
| Iodine nutrition post-intervention |  |  |  |  |  |  |  |
| UIC, *µg/L* |  |  |  |  |  |  |  |
| <p33 (57 *µg/L)* | 1.1 (0.8-1.3) | 0.430 | 15.4 (14.6-16.1) | 0.220 | 4.4 (3.8-4.6) | 0.085 | 1 (2) |
| p33-p66 (57-130 *µg/L)* | 1.1 (0.7-1.6) |  | 15.5 (14.3-16.0) |  | 4.3 (4.9-4.9) |  | 1 (2) |
| >p66 (130 *µg/L)* | 1.2 (0.9-1.6) |  | 14.7 (13.6-15.9) |  | 4.6 (4.3-4.8) |  | 0 |
| Estimted iodine intake^c^ *µg/day* |  |  |  |  |  |  |  |
| <p33 (149 *µg/day)* | 1.1 (0.8-1.3) | 0.432 | 15.3 (14.0-16.1) | 0.820 | 4.4 (4.1-5.0) | 0.322 | 0 |
| p33-p66 (149-230 *µg/day)* | 1.1 (0.8-2.0) |  | 15.3 (13.1-16.1) |  | 4.4 (3.9-4.6) |  | 1 (2) |
| >p66 (230 *µg/day)* | 1.2 (0.9-1.6) |  | 14.9 (14.4-15.8) |  | 4.7(4.3-4.8) |  | 1 (2) |
| Tg *µg/L* |  |  |  |  |  |  |  |
| <p33 (10.8 *µg/L)* | 1.0 (0.8-1.4) | 0.169 | 15.3 (14.0-15.8) | 0.687 | 4.5 (4.0-5.0) | 0.169 | 0 |
| p33-p66 (10.8-22.0 *µg/L)* | 1.2 (0.8-1.4) |  | 15.4 (14.5-16.2) |  | 4.5 (4.4-4.8) |  | 1 (2) |
| >p66 (22.0 *µg/L)* | 1.2 (0.9-1.9) |  | 14.9 (13.5-16.0) |  | 4.2 (3.8-4.6) |  | 1 (2) |

Values are given as median (p25-p75) and n (%) of the total sample (n=45). ^a^ Differences tested with Mann Whitney U test between p33 and p66. ^b^Eq.2. ^c^Eq.3

**Table S4** TSH, fT4 and fT3 post-intervention in different groups of iodine status and estimated iodine intake pre-intervention.

|  | TSH post-intervention  *mIU/L* (n=41) | p ^a^ | fT4 post-intervention  *pmol/L* (n=41) | p ^a^ | fT3 post-intervention  *pmol/L* (n=41) | p ^a^ | TPOAb positive  post-intervention (n=41) |
| --- | --- | --- | --- | --- | --- | --- | --- |
| Iodine nutrition pre-intervention |  |  |  |  |  |  |  |
| UIC, *µg/L* |  |  |  |  |  |  |  |
| <p33 (200 *µg/L)* | 1.2 (0.9-1.4) | 0.928 | 15.6 (14.7-16.0) | 0.294 | 4.4 (3.9-4.8) | 0.555 | 1 (2) |
| p33-p66 (200-490 *µg/L)* | 0.9 (0.7-1.9) |  | 15.4 (14.1-16.3) |  | 4.6 (4.3-5.0) |  | 1 (2) |
| >p66 (490 *µg/L)* | 1.2 (0.8-1.6) |  | 14.5 (13.4-15.9) |  | 4.5 (4.2-4.6) |  | 0 |
| Estimted iodine intake^c^ *µg/day* |  |  |  |  |  |  |  |
| <p33 (407 *µg/day)* | 1.1 (0.9-1.4) | 0.352 | 15.7 (15.1-16.2) | 0.227 | 4.5 (4.1-4.8) | 0.910 | 0 |
| p33-p66 (407-1155 *µg/day)* | 0.8 (0.6-1.2) |  | 14.2 (12.5-15.5) |  | 4.4 (4.0-5.0) |  | 1 (2) |
| >p66 (1155 *µg/day)* | 1.3 (0.9-1.8) |  | 15.1 (14.0-16.3) |  | 4.5 (4.1-4.6) |  | 1 (2) |
| Tg *µg/L* |  |  |  |  |  |  |  |
| <p33 (12.7 *µg/L)* | 1.0 (0.8-1.4) | 0.467 | 15.2 (14.1-15.7) | 0.821 | 4.5 (4.2-5.0) | 0.058 | 0 |
| p33-p66 (12.7-21.4 *µg/L)* | 1.2 (0.9-1.4) |  | 15.7 (13.5-16.5) |  | 4.5 (4.4-4.8) |  | 0 |
| >p66 (21.4 *µg/L)* | 1.1 (0.7-1.7) |  | 14.9 (13.9-15.7) |  | 4.2 (3.7-4.6) |  | 2 (5) |

Values are given as median (p25-p75) and n (%) of the total sample (n=45). ^a^ Differences tested with Mann Whitney U test between p33 and p66. . ^b^Eq.2. ^c^Eq.3

**Table S5** Iodine nutrition pre-intervention in tertiles of participants with decrease in TSH from pre to post-intervention.

|  | UIC *µg/L* | p ^a^ | Estimated iodine intake^c^ *µg/day* | p ^a^ | Tg *µg/L* | p ^a^ |
| --- | --- | --- | --- | --- | --- | --- |
| Decrease in TSH from pre to post *mIU/L* (n=34) |  |  |  |  |  |  |
| <p33 (0.4) *mIU/L* | 200 (67-320) | 0.004 | 395 (163-655) | 0.002 | 16.2 (6.7-31.0) | 0.034 |
| p33-p66 (0.4-0.9 *mIU/L)* | 340 (148-1575) |  | 1183 (486-1997) |  | 13.1 (10.1-25.8) |  |
| >p66 (0.9 *mIU/L)* | 1000 (250-1800) |  | 2454 (667-3382) |  | 35.0 (16.4-62.0) |  |
| Increase in TSH from pre to post *mIU/L* (n=7) | 86 (45-391) |  | 322 (317-623) |  | 18.6 (6.0-20.2) |  |

Values are given as median (p25-p75). ^a^ Differences tested with Mann Whitney U test between p33 and p66. . ^b^Eq.2. ^c^Eq.3.
